# Brain Frequency-Specific Changes in the Spontaneous Neural Activity Are Associated With Cognitive Impairment in Patients With Presbycusis

**DOI:** 10.3389/fnagi.2021.649874

**Published:** 2021-07-14

**Authors:** Fuxin Ren, Wen Ma, Wei Zong, Ning Li, Xiao Li, Fuyan Li, Lili Wu, Honghao Li, Muwei Li, Fei Gao

**Affiliations:** ^1^Department of Radiology, Shandong Provincial Hospital, Cheeloo College of Medicine, Shandong University, Jinan, China; ^2^Department of Radiology, Shandong Provincial Hospital Affiliated to Shandong First Medical University, Jinan, China; ^3^Department of Otolaryngology, The Central Hospital of Jinan City, Cheeloo College of Medicine, Shandong University, Jinan, China; ^4^CAS Key Laboratory of Mental Health, Institute of Psychology, Chinese Academy of Sciences, Beijing, China; ^5^Department of Neurology, Shandong Provincial Hospital Affiliated to Shandong First Medical University, Jinan, China; ^6^Vanderbilt University Institute of Imaging Science, Nashville, TN, United States

**Keywords:** presbycusis, cognitive impairment, low-frequency fluctuation, frequency band, functional connectivity

## Abstract

Presbycusis (PC) is characterized by preferential hearing loss at high frequencies and difficulty in speech recognition in noisy environments. Previous studies have linked PC to cognitive impairment, accelerated cognitive decline and incident Alzheimer’s disease. However, the neural mechanisms of cognitive impairment in patients with PC remain unclear. Although resting-state functional magnetic resonance imaging (rs-fMRI) studies have explored low-frequency oscillation (LFO) connectivity or amplitude of PC-related neural activity, it remains unclear whether the abnormalities occur within all frequency bands or within specific frequency bands. Fifty-one PC patients and fifty-one well-matched normal hearing controls participated in this study. The LFO amplitudes were investigated using the amplitude of low-frequency fluctuation (ALFF) at different frequency bands (slow-4 and slow-5). PC patients showed abnormal LFO amplitudes in the Heschl’s gyrus, dorsolateral prefrontal cortex (dlPFC), frontal eye field and key nodes of the speech network exclusively in slow-4, which suggested that abnormal spontaneous neural activity in PC was frequency dependent. Our findings also revealed that stronger functional connectivity between the dlPFC and the posterodorsal stream of auditory processing, as well as lower functional coupling between the PCC and key nodes of the DMN, which were associated with cognitive impairments in PC patients. Our study might underlie the cross-modal plasticity and higher-order cognitive participation of the auditory cortex after partial hearing deprivation. Our findings indicate that frequency-specific analysis of ALFF could provide valuable insights into functional alterations in the auditory cortex and non-auditory regions involved in cognitive impairment associated with PC.

## Introduction

The prevalence of presbycusis (PC), referred to as age-related hearing loss, is nearly 45% of elderly people > 60 years and 65% of elderly people > 75 years (Cruickshanks et al., 1998). PC most often show a decline of hearing ability toward high frequencies, which play a crucial role in speech recognition (Gates and Mills, 2005). Recently, an increasing number of studies have linked PC to cognitive impairment, accelerated cognitive decline and incident Alzheimer’s disease (Panza et al., 2015; Loughrey et al., 2018; Ren et al., 2019). A longitudinal research demonstrated that elderly people with mild hearing decline had a twofold increased risk, and those with moderate hearing decline had a threefold increased risk of developing dementia compared to those without hearing loss (Lin et al., 2011a), and PC was recently identified as potentially the most modifiable risk factor in dementia such as Alzheimer’s disease (Livingston et al., 2017, 2020; Chern and Golub, 2019). However, the neural mechanisms of cognitive impairment in patients with PC remain unclear.

Previous magnetic resonance imaging (MRI) studies have demonstrated that the auditory cortex and cognition-related cortical regions are involved in cognitive impairment in patients with PC (Gao et al., 2015; Ma et al., 2016; Ren et al., 2018; Chen et al., 2020; Delano et al., 2020). In structural MRI studies using surface-based morphometry, compared to age-matched normal hearing controls, patients with PC showed decreased gray matter (GM) volume or thickness in auditory region, precuneus and posterior cingulate cortex (PCC), which are core nodes of default mode network (DMN) (Ren et al., 2018), as well as anterior cingulate cortex (ACC) and parahippocampus (Belkhiria et al., 2019). Interestingly, 18F-fluro-deoxyglucose (FDG) positron Emission Tomography (PET) has found decreased cerebral metabolism in the right auditory cortex and increased metabolism in the left inferior parietal gyrus of late-onset deaf patients, which was positively associated with better cognitive performance (Verger et al., 2017). More recently, an arterial spin labeling (ASL) MRI study found the cerebral blood flow of the right auditory cortex was significantly reduced, which was negatively associated with the audiogram steepness in patients with PC (Ponticorvo et al., 2019). Resting-state functional MRI (rs-fMRI) has become a valuable technique to explore neuronal fluctuations in GM in a number of neurologic and psychiatric diseases. For example, a rs-fMRI study suggested that the functional connectivity (FC) between hippocampus and inferior parietal lobule (IPL) were significantly correlated Trail-Making Test B (TMT-B) scores in patients with PC (Chen et al., 2020). Additionally, amplitude of low-frequency fluctuations (ALFF) was decreased in the superior temporal gyrus (STG) and precuneus in patients with PC (Chen et al., 2018), which demonstrated that changes of blood oxygen level-dependent (BOLD) signal intensity within specific brain regions (Zang et al., 2007).

The abovementioned rs-fMRI studies have explored low-frequency oscillations (LFO) connectivity or amplitude of PC-related neural activity in a frequency band (0.01–0.1 Hz). However, several recent studies have demonstrated that distinct frequency bands sensitively reveal changes of spontaneous neural activity (Penttonen, 2003; Mantini et al., 2007). Specifically, there are four frequency bands in LFO: slow-5 (0.01–0.027 Hz), slow-4 (0.027–0.073 Hz), slow-3 (0.073–0.198 Hz), and slow-2 (0.198–0.25 Hz) (Zuo et al., 2010). The slow-4 and slow-5 frequency bands mainly indicate GM-related LFO amplitudes (Zuo et al., 2010), whereas the slow-2 and slow-3 frequency bands mainly reflect white matter-related LFO amplitudes and physiological noises (Zuo et al., 2010; Baria et al., 2011). Frequency-dependent changes of spontaneous neural activity have been explored in a number of neurologic and psychiatric diseases, whereas it remains unclear whether the abnormalities occur in patients with PC. Moreover, ALFF reflects brain activity by BOLD fluctuation amplitude within specific brain regions (Zuo et al., 2010), whereas FC is often calculated the temporal correlation of BOLD signal between brain regions (Biswal et al., 1995). Local and remote spontaneous neural activity can be observed using these two parameters in a complementary way (Qi et al., 2015). However, to date there is no study detecting changes of spontaneous neural activity in PC patients using ALFF and FC values together.

In this study, therefore, we applied ALFF at slow-4 and slow-5 frequency bands to explore changes of LFO amplitudes in patients with PC. Then, regions showing altered ALFF were defined as seeds to detect FC which reflects the temporal correlation of these regions with other regions. Based on previous findings, we hypothesized that (1) abnormal ALFF may be frequency dependent in patients with PC, (2) these frequency-dependent changes of spontaneous neural activity (if it is altered) may be associated with PC-related cognitive impairment, and (3) these changes in LFO amplitude would be related to changes in FC.

## Materials and Methods

### Subjects

This study included 102 subjects: fifty-one PC patients (PC group, 28 males/23 females, mean age, 65.16 ± 2.43 years) and fifty-one age-, sex- and education-level matched normal hearing controls (NH group, 21 males/30 females, mean age, 64.67 ± 1.67 years) (Table 1). All participants were Chinese Han nationality with right handedness (Gong et al., 2005; Hatta, 2007). Hearing loss was assessed by the speech-frequency pure tone average (PTA) of thresholds at 0.5, 1, 2, and 4 kHz, while the PTA value of 25 dB HL was accepted as the normal hearing threshold limit (Lin et al., 2011b). This work was approved by the Shandong University Institutional Review Board, and the written informed consent was obtained from each participant.

**TABLE 1:** Subjects’ demographic and clinical data.

**Characteristics**	**PC group**	**NH group**	***P*-value**
	**(*n* = 51)**	**(*n* = 51)**	
Gender (male/female)	28/23	21/30	0.165
Age (years)	65.16 ± 2.43	64.67 ± 1.67	0.238
Education (years)	10.31 ± 4.37	11.43 ± 2.56	0.119
Disease duration (years)	5.80 ± 4.90	–	–
Diabetes (yes/no)	8/43	8/43	1.000
Smoking (yes/no)	9/42	4/47	0.138
Alcohol abuse (yes/no)	4/47	2/49	0.674
Hypertension (yes/no)	27/24	20/31	0.164
Hyperlipemia (yes/no)	9/42	9/42	1.000
Anxiety	3.04 ± 3.19	3.61 ± 3.38	0.384
Depression	3.84 ± 3.89	3.33 ± 3.52	0.489
PTA (dB/HL)	38.33 ± 12.23	10.83 ± 3.50	** < 0.001**
SRT (dB/HL)	38.34 ± 13.56	11.01 ± 3.97	** < 0.001**
MoCA	23.53 ± 4.90	26.67 ± 2.90	**<0.001**
AVLT	48.22 ± 9.96	51.71 ± 13.08	0.133
SDMT	24.80 ± 12.23	35.33 ± 10.99	**<0.001**
Stroop (s)	153.25 ± 43.60	132.61 ± 27.21	**0.005**
TMT-A (s)	77.25 ± 38.42	59.90 ± 26.41	**0.009**
TMT-B (s)	212.16 ± 93.84	159.63 ± 62.87	**0.001**

*The data are presented as means ± standard deviations. Values in bold are statistically significant.*

*PTA, pure tone average; SRT, speech reception threshold; PC, presbycusis; NH, normal hearing controls; MoCA, Montreal Cognitive Assessment; AVLT, Auditory Verbal Learning Test; SDMT, Symbol Digit Modalities Test; TMT, Trail-Making Test; levels of anxiety and depression were assessed according to the Hospital Anxiety and Depression Scale (HADS).*

Inclusion criteria for the PC group were PTA > 25 dB HL in the better hearing ear and age ≥ 60 years; and for the NH group, it was PTA ≤ 25 dB HL in the better hearing ear. Exclusion criteria for both PC and the NH groups were as follows: (1) ear diseases that affect hearing thresholds and sensorineural hearing losses other than PC; (2) asymmetrical hearing loss or conductive hearing loss; (3) Meniere’s disease, acoustic neuroma, hyperacusis and tinnitus; (4) neurological or psychiatric disease; (5) MRI contraindications (e.g., claustrophobia, pacemakers, and metal implants).

### Auditory and Cognitive Function Test

The tympanometry, pure tone audiometry, and speech reception threshold (SRT) were conducted using Madsen Electronics Zodiac 901, Madsen Electronics Midimate 622 and HOPE software, respectively. Air conduction was measured at 0.125–8 kHz, and SRT was tested according to the American Speech-language Hearing Association. Details of SRT acquisition procedures are provided in the Supplementary Materials. The cognitive status of PC patients and controls was assessed using the Montreal Cognitive Assessment (MoCA) (Galea and Woodward, 2005; Nasreddine et al., 2005), Auditory Verbal Learning Test (AVLT, Chinese version) (Zhao et al., 2012), Symbol Digit Modalities Test (SDMT) (Van Schependom et al., 2014), Stroop color word interference test (Savitz and Jansen, 2003), and Trail-Making Test (including both the TMT-A and TMT-B) (Sanchez-Cubillo et al., 2009). Finally, depression and anxiety status of PC patients and controls were assessed by the Hospital Anxiety and Depression Scale (HADS) (Zigmond and Snaith, 1983).

### MRI Acquisition

All subjects were scanned with a 3.0 T scanner (Philips, Achieva TX) using an eight-channel phased-array head coil. The scanning sessions included: (i) localization, (ii) 5-min three-dimensional (3D) T1-weighted images, (iii) 8-min rs-fMRI, (iv) 2-min fluid-attenuated inversion recovery (FLAIR) sequence. The T1-weighted images were acquired using 3D turbo field echo sequence: time to repetition time (TR) = 8.1 ms; time to echo time (TE) = 3.7 ms; voxel size = 1 × 1 × 1 mm^3^; field of view = 24 × 24 cm^2^; slice thickness = 1 mm; 160 slices. The rs-fMRI data were acquired using an echo-planar gradient echo pulse sequence: TR = 2,000 ms; TE = 35 ms; field of view = 24 × 24 cm^2^; in-plane resolution = 3.75 × 3.75 mm^2^; slice thickness = 4 mm; 35 slices; 240 volumes. FLAIR images were used to evaluate white matter hyperintensity and intracranial structural lesions.

### Functional Data Preprocessing

Functional data preprocessing was conducted with the Data Processing & Analysis for Brain Imaging (DPABI) V5.1 toolbox (Yan et al., 2016) based on Statistical Parametric Mapping (SPM) 12 software^1^. Preprocessing for each subject included (1) the removal of the first 10 volumes of each fMRI scan to avoid the influence of image signal fluctuation at the beginning of scanning; (2) slice-time and head motion corrected; (3) coregistration of T1 and fMRI image; (4) normalization of T1 to Montreal Neurological Institute (MNI) space and using the resulting deformation fields to project the functional images to MNI space; (5) nuisance covariate regression with the Friston 24 head motion parameters (Friston, 2015) and cerebrospinal fluid signal (Ashburner and Friston, 2005); (6) isotropic smoothing Gaussian kernel of 4 mm full width at half maximum; (7) linear detrending.

### ALFF Calculation and FC Analysis

Firstly, the ALFF values at the slow-5 (0.01–0.027 Hz) and slow-4 (0.027–0.073 Hz) frequency bands were calculated by the DPABI toolbox. The time series for a given voxel was converted to the frequency domain using Fast Fourier Transform, then the square root of the power spectrum was computed and then averaged at each voxel, and this averaged square root was defined as the ALFF. Secondly, preprocessed rs-fMRI images were bandpass filtered at the slow-5 and slow-4 frequency bands. Regions showing altered ALFF between the PC and NH groups were defined as seeds to detect FC. Specifically, correlation analysis of time course was conducted between the seeds and all other brain voxels in the PC and NH groups.

### Statistical Analysis

Group differences in demographic and auditory and cognitive function scores were evaluated by the two-tailed *t*-test, and group differences in sex, diabetes, smoking, alcohol abuse, hypertension, and hyperlipemia were assessed by the chi-square test in PASW 17.0 software (Chicago, IL, United States). *P*-values of less than 0.05 were accepted as significant.

Two-way repeated-measures analysis of variance (ANOVA) was conducted to assess the main effects of group and frequency band, and their interactions in ALFF, group (PC vs. NH) served as a between-subject factor; frequency band (slow-5 vs. slow-4) served as a repeated-measures factor. Age, sex, and education levels were imported as covariates. The FDR correction (*p* < 0.01, cluster size > 5 voxels) was used to correct the T map for main effects and F map for interaction effect using SPM 12. Then, differences between the PC and NH groups in each frequency band were assessed by the *post hoc* two-sample *t*-tests. The FDR correction (*p* < 0.01, cluster size > 5 voxels) was used to correct the T map using SPM 12. If these results cannot survive after FDR correction, the AlphaSim-corrected (*p* < 0.05, cluster size > 19 voxels) was used to correct using REST V1.8 (Song et al., 2011).

For following seed-based FC analysis, a two-sample *t*-test was used to compare the FC maps between the PC and NH groups in the slow-4 and slow-5 band, respectively. The significance level was set at an FDR-corrected *p* < 0.05, cluster size > 20 voxels. Finally, the relationships in the PC and NH groups between the mean ALFF or FC value within regions that exhibited significant group differences and cognitive function or audiological outcomes (controlled for age, sex, education level) were assessed using partial correlation analyses.

## Results

### Demographic and Clinical Characteristics

**FIGURE 1:**
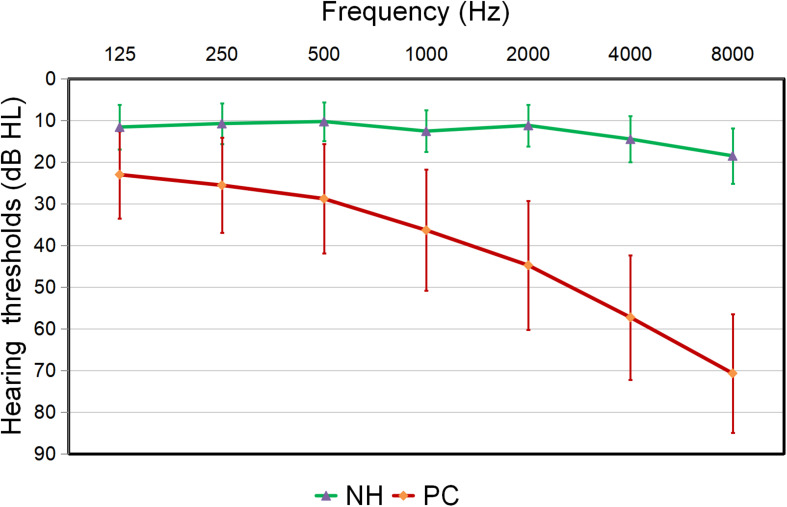
Hearing thresholds of the presbycusis (PC) and normal hearing controls (NH) groups (means ± standard deviation) in air conduction. Hearing thresholds from both ears are averaged.

Compared to the controls, PC patients performed worse on the MoCA, SDMT, Stroop, TMT-A, TMT-B tests, PTA, and SRT (*p* < 0.05, Table 1). No subject was excluded for severe white matter hyperintensity or intracranial lesions. Figure 1 shows the average hearing thresholds of the PC and NH groups, respectively. The results of correlations between cognitive function and PTA or SRT in the PC and NH groups were added to the Supplementary Materials.

### ALFF Results

**FIGURE 2:**
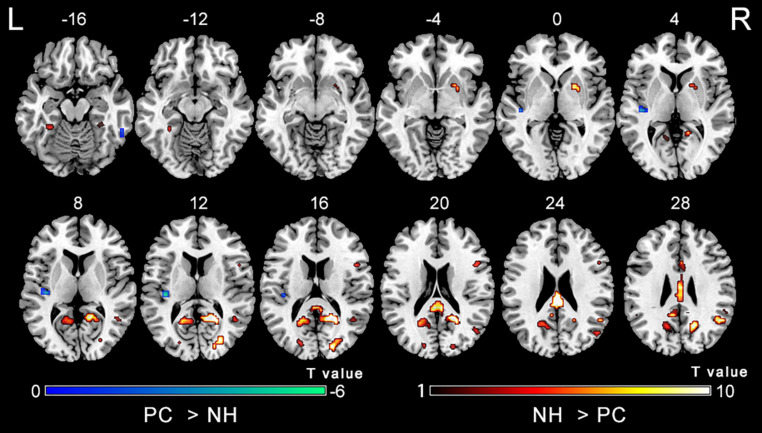
Main effect of the group factor on ALFF. Hot and cold colors indicate significantly higher and lower ALFF in the presbycusis (PC) group than in the normal hearing controls (NH) group, respectively. Results obtained by a two-way repeated-measures ANOVA. FDR corrected *p* < 0.01, cluster size > 5 voxels. L, left; R, right; ALFF, amplitude of low-frequency fluctuation; ANOVA, analysis of variance.

Figure 2 shows main effects of group from the two-way repeated-measure ANOVA. Compared with the NH group, the PC group showed significantly decreased ALFF in the bilateral posterior cingulate cortex (PCC), precuneus, superior occipital gyrus (SOG), angular gyrus (AG), frontal eye field (FEF), paracentral lobule and supplementary motor area (SMA); the right superior marginal gyrus (SMG). Compared with the NH group, the PC group showed significantly increased ALFF in the bilateral inferior temporal gyrus (ITG) and the left Heschl’s gyrus (HG). The related results without covariates factors were added to Supplementary Materials.

**FIGURE 3:**
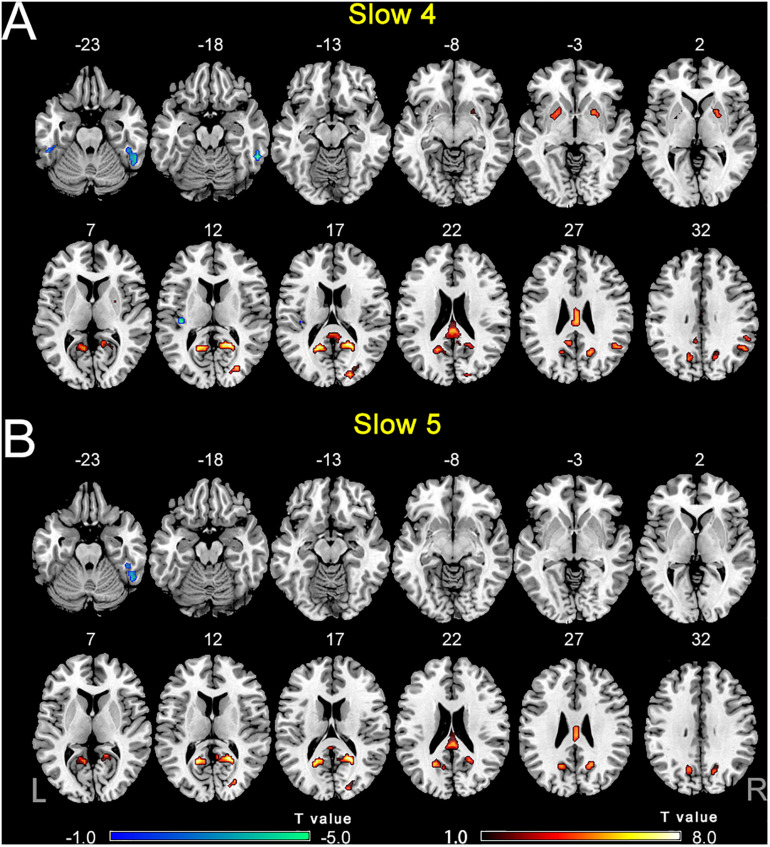
**(A)** The difference of ALFF between the presbycusis (PC) and normal hearing controls (NH) groups in slow-4. **(B)** The difference of ALFF between the PC and NH groups in slow-5. Hot and cold colors indicate significantly higher and lower ALFF in the PC group than in the NH group, respectively. Results obtained by a two-sample *t*-test. FDR corrected *p* < 0.01, cluster size > 5 voxels. L, left; R, right; ALFF, amplitude of low-frequency fluctuation.

The comparisons between PC and NH groups showed some similarities in the two frequency bands, such as decreased ALFF in the bilateral precuneus; the right PCC and SOG, as well as increased ALFF in the right ITG in the PC patients in both bands (Figure 3 and Table 2). In contrast, some obvious differences also existed between the two bands. There was decreased ALFF in the bilateral putamen; the right AG, SMG, and FEF; the left PCC, Inferior Parietal Gyrus (IPG), dlPFC and SMA, along with increased ALFF in the left HG and ITG in PC patients compared to the controls in slow-4 band, changes which were not seen in the slow-5 band (Figure 3 and Table 2). The results between the PC and NH groups in full band (0.01–0.1 Hz) were added to the Supplementary Materials.

**TABLE 2:** The difference of ALFF in each frequency band between PC and NH groups.

**Fequency band**	**Brain region**	**Brodmann area**	**MNI coordinates**			***T*-value**	**Cluster size**
			**x**	**y**	**z**		
Slow-4 (0.027–0.073 Hz)							
PC > NH							
	R inferior temporal gyrus	20	42	−39	−30	−5.4433	45
	L inferior temporal gyrus	20	−48	−39	−2	−4.4161	8
	L heschl gyrus	41, 42	−39	−24	15	−5.0421	6
NH > PC							
	R putamen	—	24	9	−6	5.2885	26
	L putamen	—	−21	6	−3	4.5976	18
	L precuneus	7	−18	−54	18	7.9703	90
	R posterior cingulate cortex	23	21	−51	18	8.6013	127
	R superior occipital gyrus	18	21	−87	18	5.8254	28
	R angular gyrus	39	51	−57	36	6.1707	32
	L Posterior Cingulate Cortex	31	−6	−48	27	4.5817	13
	R precuneus	7	18	−63	30	5.8248	18
	R superior marginal gyrus	40	54	−45	39	5.9243	22
	L inferior parietal gyrus	39	−36	−60	42	5.1904	14
	L dorsolateral prefrontal cortex	46	−36	27	42	5.2038	11
	R frontal eye field	8	21	30	48	6.2324	22
	L supplementary motor area	6	−12	6	63	5.6375	34
Slow-5 (0.01–0.027 Hz)							
PC > NH							
	R inferior temporal gyrus	20	51	−48	−24	−5.1617	32
NH > PC							
	L Precuneus	7	−18	−54	15	7.8129	93
	R Precuneus	7	21	−51	12	9.3104	78
	R superior occipital gyrus	18	21	−84	15	5.7463	15
	R posterior cingulate cortex	23	3	−33	24	6.4022	45
	R supplementary motor area	6	−6	12	57	5.2753	35

*FDR corrected p < 0.01, cluster size > 5 voxels. ALFF, amplitude of low-frequency fluctuation; PC, presbycusis; NH, normal hearing controls; MNI, Montreal Neurological Institute; L, left; R, right.*

**FIGURE 4:**
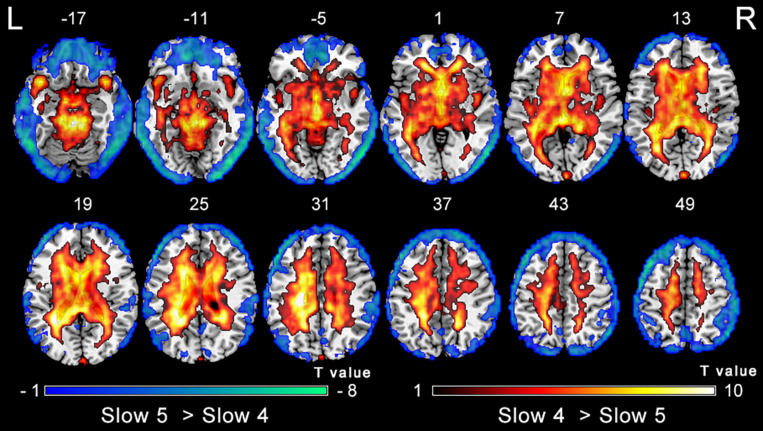
Main effect of the frequency band factor on ALFF. Hot and cold colors indicate significantly higher and lower ALFF in the slow-4 band than in the slow-5 band, respectively. Results obtained by a two-way repeated-measures ANOVA. FDR corrected *p* < 0.01, cluster size > 5 voxels. L, left; R, right; ALFF, amplitude of low-frequency fluctuation; ANOVA, analysis of variance.

**FIGURE 5:**
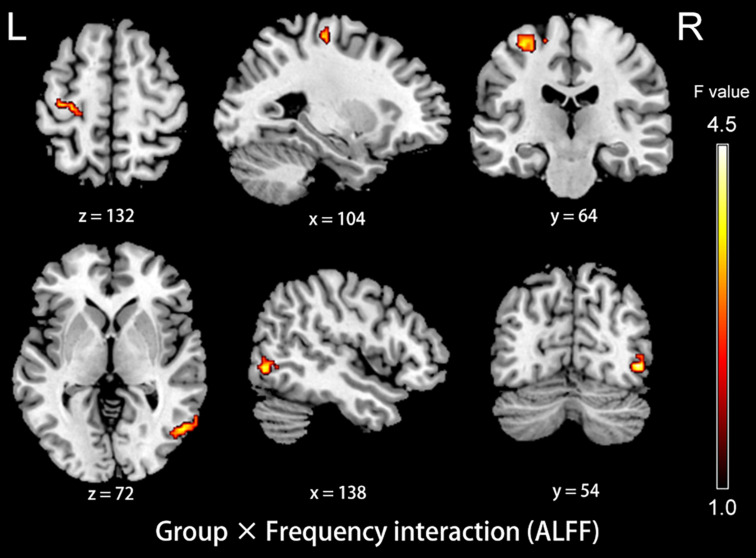
The interaction between group and frequency band on ALFF. Hot color indicates that decreased ALFF in the left precentral gyrus and the right middle temporal gyrus in presbycusis compared to controls was greater in slow-4 than those in slow-5. The results were obtained by a two-way repeated-measures ANOVA and *post hoc t*-tests. AlphaSim corrected (*p* < 0.05, cluster size > 19 voxels). L, left; R, right; ALFF, amplitude of low-frequency fluctuation; ANOVA, analysis of variance.

The analysis on the effect of frequency band in both groups showed that the ALFF in the slow-4 band compared to that in the slow-5 band was higher in the pons, midbrain, caudate, putamen, thalamus, SMA, hippocampus, and cerebellum, but lower in the MTG, ITG, inferior occipital gyrus, precentral gyrus, middle frontal gyrus, and superior frontal gyrus (Figure 4). The results regarding the interaction effect cannot survive after FDR correction so that we show the trends of such differences that were corrected by the AlphaSim approach instead. There was significant interaction between frequency band and group in the left precentral gyrus and the right MTG, and decreased ALFF in the two regions in PC patients compared to controls was greater in the slow-4 band than those in the slow-5 band (Figure 5 and Table 3). The percent amplitude of fluctuation (PerAF) (Jia et al., 2017; Zhao et al., 2018; Yu et al., 2019) was also applied to explore changes of LFO amplitudes in patients with PC. The related results were added to the Supplementary Materials.

**TABLE 3:** Brain regions showing significant interaction in the ALFF between group and frequency band.

**Brain region**	**Brodmann area**	**MNI coordinates**			***F*-value**	**Cluster size**
		**x**	**y**	**z**		
R middle temporal gyrus	21	45	−72	−3	4.4458	45
L precentral gyrus	4	−33	−21	60	3.8567	26

*AlphaSim corrected (p < 0.05, cluster size > 19 voxels). ALFF, amplitude of low-frequency fluctuation; MNI, Montreal Neurological Institute; L, left; R, right.*

### Functional Connectivity Results

**FIGURE 6:**
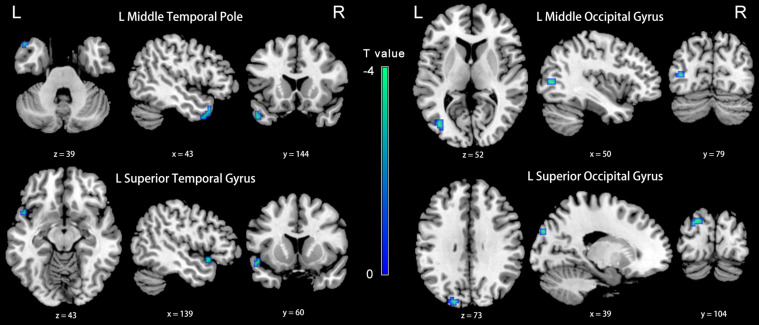
Between-group differences in FC analyses of the seed of left dlPFC in slow-4. Winter color indicates significantly higher FC in the presbycusis (PC) group than in the normal hearing controls (NH) group. Results obtained by a two-sample *t*-test. *p* < 0.001 (uncorrected, cluster size > 5 voxels). L, left; R, right; ALFF, amplitude of low-frequency fluctuation; FC, functional connectivity; dlPFC, dorsolateral prefrontal cortex.

Compared with the NH group, the dlPFC in the slow-4 band showed stronger FC with the left middle temporal pole, STG, middle occipital gyrus (MOG) and SOG in patients with PC (*p* < 0.001, uncorrected) (Figure 6 and Table 4). However, none of these differences can survive after FDR correction (*p* < 0.05). The related results without covariates factors were added to the Supplementary Materials.

**TABLE 4:** Brain regions showing significantly different functional connectivity of dlPFC in the slow-4 band in PC group.

**Brain region**	**Brodmann area**	**MNI coordinates**			***T*-value**	**Cluster size**
		**x**	**y**	**z**		
L middle temporal pole	38	−45	18	−30	−3.5614	13
L superior temporal gyrus	22	−48	12	−12	−3.8103	6
L middle occipital gyrus	—	−39	−75	6	−3.9123	13
L superior occipital gyrus	19	−27	−87	30	−3.8953	15

*p < 0.001 (uncorrected, cluster size > 5 voxels). PC, presbycusis; MNI, Montreal Neurological Institute; L, left; R, right.*

**FIGURE 7:**
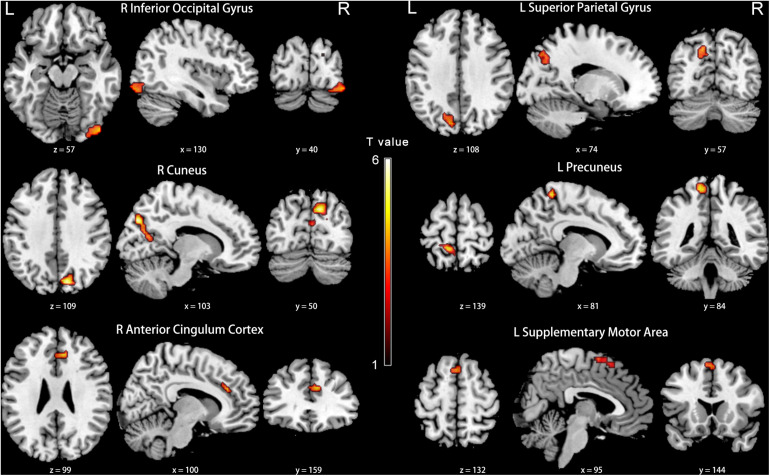
Between-group differences in FC analyses of the seed of right PCC in slow-5. Hot color indicates significantly lower FC in the presbycusis (PC) group than in the normal hearing controls (NH) group. Results obtained by a two-sample *t*-test. FDR corrected *p* < 0.05, cluster size > 20 voxels. L, left; R, right; ALFF, amplitude of low-frequency fluctuation; FC, functional connectivity; PCC, posterior cingulate cortex.

Compared with the NH group, the PCC in the slow-5 band showed weaker FC with the right inferior occipital gyrus, cuneus, anterior cingulate cortex (ACC), and the left superior parietal gyrus, precuneus and SMA in patients with PC (FDR corrected *p* < 0.05, cluster size > 20 voxels) (Figure 7 and Table 5).

**TABLE 5:** Brain regions showing significantly different functional connectivity of PCC in the slow-5 band in PC group.

**Brain region**	**Brodmann area**	**MNI coordinates**			***T*-value**	**Cluster size**
		**x**	**y**	**z**		
R Inferior Occipital Gyrus	18	45	−84	−15	4.6042	71
R Cuneus	7	12	−78	36	5.9099	205
R Anterior Cingulate Cortex	32	21	27	30	5.5348	51
L Superior Parietal Gyrus	7	−18	−75	42	4.2971	58
L Precuneus	—	−12	−42	66	4.9208	48
L Supplementary Motor Area	6	0	18	60	4.0550	38

*FDR corrected p < 0.05, cluster size > 20 voxels.*

*PC, presbycusis; MNI, Montreal Neurological Institute; L, left; R, right.*

### Brain-Behavior Relationships

**FIGURE 8:**
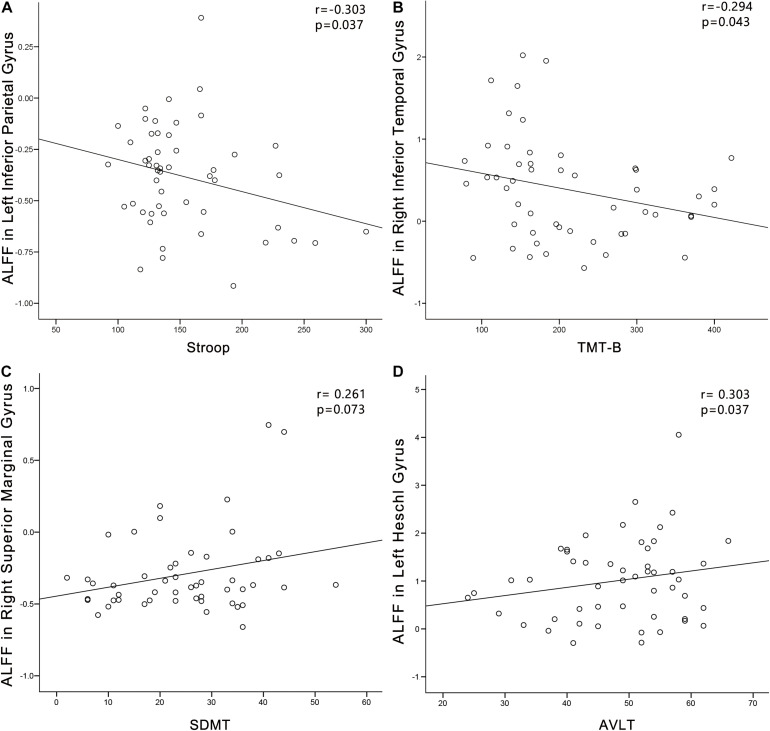
Correlations between ALFF changes and cognitive impairments in the presbycusis (PC) group. Correlations were controlled for age, sex, and education. **(A)** Stroop scores were negatively correlated with the ALFF of the left inferior parietal gyrus (*r* = −0.303, *p* = 0.037). **(B)** TMT-B scores were negatively correlated with the ALFF of the left inferior temporal gyrus (*r* = −0.294, *p* = 0.043). **(C)** SDMT scores were positively associated with the ALFF of the right superior marginal gyrus (*r* = 0.261, *p* = 0.073). **(D)** AVLT scores were positively associated with the ALFF of the left HG (*r* = 0.303, *p* = 0.037). ALFF, amplitude of low-frequency fluctuation; AVLT, Auditory Verbal Learning Test; SDMT, Symbol Digit Modalities Test; TMT, Trail-Making Test.

In the PC group (Figure 8), partial correlation analyses revealed that Stroop scores were negatively correlated with the ALFF of the left IPG (*r* = −0.303, *p* = 0.037); TMT-B scores were negatively correlated with the ALFF of the left ITG (*r* = −0.294, *p* = 0.043); SDMT scores were positively associated with the ALFF of the right SMG (*r* = 0.261, *p* = 0.073). AVLT scores were positively associated with the ALFF of the left HG (*r* = 0.303, *p* = 0.037). In the PC group, no correlations were observed between audiological characteristics or disease duration and ALFF. In the NH group, no correlations were observed between clinical characteristics and ALFF.

**FIGURE 9:**
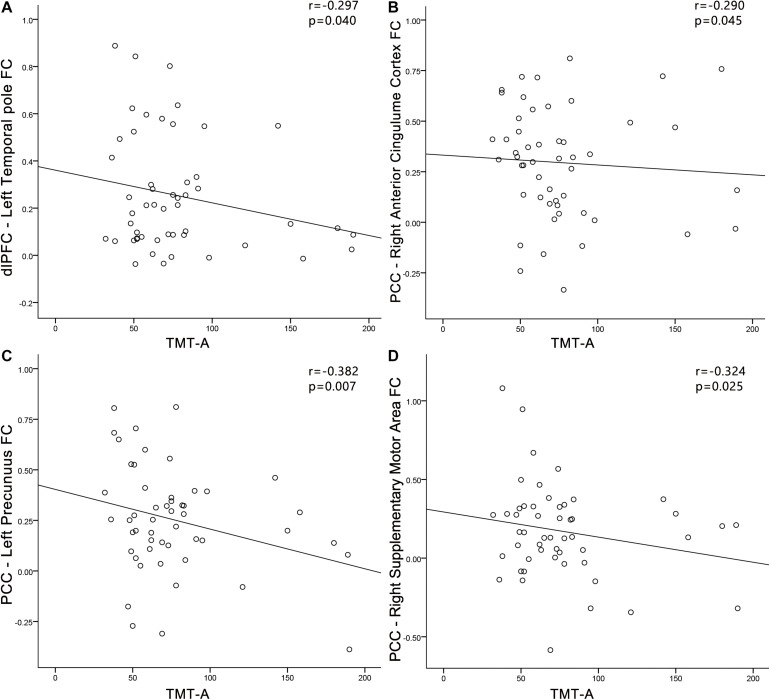
Correlations between FC changes and cognitive impairments in the presbycusis (PC) group. Correlations were controlled for age, sex, and education. **(A)** TMT-A scores was negatively correlated with the FC between dlPFC and of the left temporal pole (*r* = −0.297, *p* = 0.040); **(B)** TMT-A scores was negatively associated with the FC between PCC and the right ACC (*r* = −0.290, *p* = 0.045); **(C)** TMT-A scores was negatively associated with the FC between PCC and the left precuneus (*r* = −0.382, *p* = 0.007); **(D)** TMT-A scores was negatively associated with the FC between PCC and the right SMA (*r* = −0.324, *p* = 0.025). FC, functional connectivity; dlPFC, dorsolateral prefrontal cortex; PCC, posterior cingulate cortex; TMT, Trail-Making Test.

In the PC group (Figure 9), TMT-A scores was negatively associated with the FC between dlPFC and the left temporal pole (*r* = −0.297, *p* = 0.040). In the PC group, no correlations were observed between audiological characteristics or disease duration and FC of dlPFC. In the NH group, no relationships were observed between auditory or cognitive function scores and FC of dlPFC. In the PC group (Figure 9), TMT-A scores was negatively associated with the FC between PCC and the right ACC (*r* = −0.290, *p* = 0.045), left precuneus (*r* = −0.382, *p* = 0.007), right SMA (*r* = −0.324, *p* = 0.025), respectively. In the PC group, no correlations were observed between audiological characteristics or disease duration and FC of PCC. In the NH group, no relationships were observed between auditory or cognitive function scores and FC of PCC.

## Discussion

In this study, we investigated changes in LFO amplitudes in PC at two different frequency bands (slow-4 and slow-5). In each band, PC patients showed decreased ALFF in the precuneus, PCC and SMA, as well as increased ALFF in the ITG. Exclusively in the slow-4 band, PC patients showed decreased ALFF in the putamen, SOG, AG, SMG, IPG, dlPFC, and FEF, as well as increased ALFF in the HG. Moreover, a significant interaction between group and frequency band was found in MTG and precentral gyrus. Importantly, ALFF alterations in HG, SMG, IPG, and ITG were correlated with cognitive impairments in PC patients. In addition, dlPFC and PCC that showing changed ALFF between groups showed frequency-dependent alterations in FC, which were associated with the attention and the executive control in PC patients. To the best of our knowledge, this is the first study to demonstrate that abnormal spontaneous neural activity in PC is frequency specific.

### Abnormalities of ALFF in Slow-4 and Slow-5 Bands

Our study revealed that ALFF was significantly decreased in the PCC and precuneus in both the slow-4 and slow-5 bands, as well as in the AG and IPG in the slow-4 band in patients with PC. The PCC, precuneus, AG, and IPG are the key nodes in the DMN, which is a large-scale brain network of interacting brain regions that shows increased activity at rest and plays a critical role in self-referential mental activity and cognitive processing (Raichle et al., 2001; Mantini et al., 2007). Moreover, Stroop scores, which indicates attention function, were negatively correlated with the ALFF of the IPG in PC patients in this study. Our results are consistent with a previous study using ALFF in the typical frequency band (0.01–0.08 Hz) (Chen et al., 2018). However, in that study, a small sample size of PC patients showed decreased ALFF only in the precuneus. Our results suggested that with a large sample size and by distinguishing frequency bands, more changes in LFO amplitudes in the key nodes in the DMN in PC patients can be detected.

In this study, ALFF in the PC patients significantly decreased in the SMA in both the slow-4 and slow-5 bands. Although the SMA has been consistently reported to be involved in many aspects of motor functions, including motor preparation, motor learning and motor control (Hardwick et al., 2013; Bonini et al., 2014), the SMA has also been widely investigated in studies of speech perception, auditory processing and auditory imagery. Regarding speech perception, fMRI studies have demonstrated significant activation in the SMA in response to syllables, words and sentences (Binder et al., 2008; Meltzer et al., 2010; Liebenthal et al., 2013). Moreover, such responses seemed to be modulated by the difficulty of speech processing and comprehension. Specifically, SMA activity was stronger when the speech signal was less intelligible, and comprehension was more challenging as a result of background noise and speech rate (Du et al., 2014). PC is characterized by difficulty in speech perception and comprehension in noisy environments. Thus, the changes in LFO amplitudes in the SMA may contribute to abnormal speech recognition in patients with PC and indicate that motor cross-modal reorganization arose from long-term degraded auditory input.

### Frequency-Specific Alterations in the ALFF

There were obvious differences in PC-related neural activity between the two bands. ALFF values were decreased in the putamen, SOG, AG, SMG, FEF, IPG, and dlPFC, along with increased ALFF in the HG in PC patients compared to controls in the slow-4 band; however, there were no changes in the slow-5 band. Importantly, there was a significant interaction between the frequency band and group in the precentral gyrus and the MTG, and decreased ALFF in the two regions in PC patients was greater in slow-4 band than those in slow-5 band. Our results suggest that it is useful for sensitivity investigations of PC-related neural activity to select an appropriate frequency band.

Primary auditory cortex is located within HG, which is very important for receiving information from the ascending auditory pathway and processing auditory input (Langers et al., 2007). Our previous study revealed that PC patients showed reduced cortical thickness in the left HG, and PC patients with higher PTA had lower cortical thickness in that region (Ren et al., 2018). In this study, PC patients showed increased ALFF in the left HG, exclusively in the slow-4 band. Our findings point to a possible relationship between alterations in functional and structural organizations in the deprived auditory cortex in PC patients. Increased ALFF in HG may reflect the retention of exuberant LFO amplitudes that resulted from degraded auditory input. This is consistent with previous electrophysiological studies, in which increased spontaneous activity and neural synchrony were observed in animal models of PC (Hughes et al., 2010; Ng and Recanzone, 2018). Importantly, our study revealed that the ALFF of the HG in PC patients were positively correlated with AVLT performance, which assesses verbal learning and memory. Additionally, in one previous ALFF study, a small sample size of PC patients did not show any change in HG in the typical frequency band (Chen et al., 2018). Therefore, our study demonstrates that LFO amplitude abnormalities in HG in PC are frequency dependent, and a large cohort of patients is clearly better for reliability.

In our study, ALFF in PC patients significantly decreased in the SMG and AG exclusively in the slow-4 band. The SMG and AG have been considered to play a crucial role in phonological and semantic aspects of word processing, respectively (Seghier, 2013; Deschamps et al., 2014). More specifically, previous fMRI studies demonstrated SMG activation related to phonological processing during language tasks (Deschamps et al., 2014), and AG activation for auditory stimuli during semantic tasks (Demonet et al., 1992). Our study also revealed that the ALFF of the SMG in PC patients were positively correlated with SDMT performance, which assesses psychomotor speed. Our study also observed significantly decreased ALFF in the putamen and IPG in patients with PC exclusively in slow-4, and decreased ALFF in the precentral gyrus in PC patients compared to controls was greater in slow-4 than in slow-5. Recent studies have demonstrated the involvement of the putamen in speech articulation and high-order speech functions, including speech processing and production (Vinas-Guasch and Wu, 2017). Moreover, functional neuroimaging studies have provided corroborating evidence pointing to the critical role of the IPG in speech processing and suggested a causal involvement of the precentral gyrus in speech perception (Simonyan and Fuertinger, 2015; Smalle et al., 2015). Therefore, decreased ALFF in the putamen, IPG and precentral gyrus may suggest impairment in the core speech networks in patients with PC, indicating that long-term degraded auditory input may affect speech function.

Interestingly, we also observed decreased ALFF in the dlPFC and FEF in PC exclusively in the slow-4 band. The dlPFC, a key node in the executive control network (ECN), is most typically associated with executive functions. The FEF, a key node in the dorsal attention network (DAN), which is very important for controlling of visual attention and eye movements. Previous fMRI studies have demonstrated decreased inter-network FC between the ECN or DAN and multiple sensory networks in sensorineural hearing loss (Husain et al., 2014; Luan et al., 2019). Furthermore, recent studies have shown that the prefrontal cortex receives auditory information from auditory regions and that the prefrontal cortex is involved in auditory cognition (Plakke and Romanski, 2014). Therefore, decreased ALFF in the dlPFC and FEF may suggest functional alterations in PC involving both auditory processing and higher-order cognitive functions.

### Abnormal Functional Connectivity of dlPFC and PCC

Using FC analyses with these regions that showing changed ALFF between groups as seeds, we determined that the dlPFC in the slow-4 band was associated with increased FC of selective regions in PC. These regions included the auditory regions (temporal pole and STG) and visual regions (SOG and MOG). Stronger functional connectivity (dlPFC-temporal pole) was associated with executive control in PC. Recent studies have shown that the dlPFC receives a widespread array of afferents from the sensory cortices (Romanski et al., 1999), hence, the dlPFC is very important for multisensory integration and top-down regulation (Morrone, 2010). Our findings suggest that increased FC between the dlPFC and auditory and visual regions might underlie the cross-modal plasticity and higher-order cognitive participation of the auditory cortex after partial hearing deprivation. Moreover, the STG belongs to the posterodorsal stream of auditory processing, which is important in sensorimotor integration and spatial processing (Lima et al., 2016). Higher functional coupling between the dlPFC and posterodorsal pathway may suggest that the dlPFC is very important for maintaining auditory processing in PC. Additionally, the PCC in the slow-5 band showed weaker FC with the right ACC and the left precuneus in patients with PC, which were associated with cognitive impairments in PC patients. PCC is a hub mainly for memory and attention information processing and also adjust auditory stimuli under non-optimal conditions (Leech and Sharp, 2014), and PCC, ACC and precuneus are the core regions of the DMN. Thus, our results suggest that PC may trigger a series of cognitive compensation to complete the top-down network regulation, resulting in the decreased intra-network FC of DMN. Previous fMRI studies in sensorineural hearing loss patients have demonstrated decreased intra-network FC related to DMN, including precuneus and PCC, which consistent with our results (Luan et al., 2019).

There are some limitations to our study. First, the origins of slow-4 and slow-5 bands have not been fully explored, and the combination of electrophysiological, pathological and fMRI methods could be useful for a better description of these problems in the future. Second, between-group changes in LFO amplitudes only in slow-4 and slow-5 were investigated in our study. Future studies exploring changes in LFO amplitudes in all frequency bands and their relationships with clinical characteristics may provide valuable information on the neural mechanisms that underlie PC-related cognitive impairment. Third, our primary purpose was to identify brain areas that exhibit abnormal LFO amplitudes in PC and subsequently quantify the changes of FC between these areas and all other brain voxels at different frequency bands. Therefore, instead of exploring FC regarding every area of the brain, we focused on brain areas where altered ALFF in PC patients comparing with controls.

## Conclusion

Our study revealed that abnormal spontaneous neural activity in PC was frequency dependent, which was correlated with cognitive impairments. Moreover, higher functional coupling between the dlPFC and the posterodorsal stream of auditory processing, as well as lower functional coupling between the PCC and key nodes of the DMN might underlie the cross-modal plasticity and higher-order cognitive participation of the auditory cortex after partial hearing deprivation. Taken together, our findings indicate that frequency-specific analysis of ALFF could provide valuable insights into functional alterations in the auditory cortex and non-auditory regions involved in cognitive impairment associated with PC.

## Data Availability Statement

The raw data supporting the conclusions of this article will be made available by the authors, without undue reservation.

## Ethics Statement

The studies involving human participants were reviewed and approved by Institutional Review Board of the Shandong University. The patients/participants provided their written informed consent to participate in this study.

## Author Contributions

FG designed the experiments. WM, WZ, NL, and XL carried out the experiments. FR, FG, and FL analyzed the experimental results. LW, HL, and ML assisted. FR and FG wrote the manuscript. All authors contributed to the article and approved the submitted version.

## Conflict of Interest

The authors declare that the research was conducted in the absence of any commercial or financial relationships that could be construed as a potential conflict of interest.

## Footnotes

1 http://www.fil.ion.ucl.ac.uk/spm
