# Supplementary material for: Brain Frequency-Specific Changes in the Spontaneous Neural Activity Are Associated With Cognitive Impairment in Patients With Presbycusis
Source: Front Aging Neurosci. 2021 Jul 14;13:649874. doi: 10.3389/fnagi.2021.649874 (PMC8316979; doi:10.3389/fnagi.2021.649874)
Supplement: Supplementary file 1 [file Table_1.DOCX]

Supplementary Material

Brain Frequency-specific Changes in the Spontaneous Neural Activity Are Associated with Cognitive Impairment in Patients with Presbycusis

Fuxin Ren, Wen Ma_,_ Wei Zong, Ning Li, Xiao Li, Fuyan Li, Lili Wu, Honghao Li, Muwei Li, Fei Gao*

*** Correspondence:**Fei Gao
feigao6262@163.com

**1. SRT test**

Speech reception threshold (SRT) was performed with a clinical audiometer (GSI AudioStar Pro) and equipped with TDH-50P headphones in quiet conditions. First, according to the value of PTA, an initial sound intensity is groped to ensure that the subject is able to exactly recognize 5 spondee words under this intensity. If the subject cannot recognize, the software will increase the initial sound intensity. The software then automatically controls sound intensity: the sound intensity reduces by 5 dB for every five words played. The test is stopped when the subject is unable to exactly recognize 5 spondee words. Finally, the number of exact recognized words are counted in the whole descending process, then the software subtracts the number of exact recognized words from initial sound intensity and adds a correction factor (2.5 dB), which is the subject's speech recognition threshold.

**2. PerAF results**

The percent amplitude of fluctuation (PerAF) (Jia et al., 2017; Zhao et al., 2018; Yang et al., 2019) was applied to explore changes of low-frequency oscillation (LFO) amplitudes in patients with presbycusis (PC). Consistent with the previous statistical model for low-frequency fluctuation (ALFF), two-way repeated-measures analysis of variance (ANOVA) was conducted to assess the main effects of group and frequency band, and their interactions in PerAF, group (PC vs. NH) served as a between-subject factor; frequency band (slow-5 vs. slow-4) served as a repeated-measures factor. Age, sex and education levels were imported as covariates. There were significant main effects of frequency band from the two-way repeated-measure ANOVA (FWE correction p < 0.05, cluster size > 5 voxels), as seen in Figure S4. However, there were no significant main effects of group from the two-way repeated-measure ANOVA, as seen in Figure S2. There were no significant between-group differences in the slow-4 or slow-5 band, as seen in Figure S3. There were no significant interactions between frequency band and group in PerAF.

**3. ALFF results without covariates factors**

Figure S5 shows main effects of group from the two-way repeated-measure ANOVA. Compared with the NH group, the PC group showed significantly decreased ALFF in the bilateral posterior cingulate cortex (PCC), precuneus, superior occipital gyrus (SOG), angular gyrus (AG), frontal eye field (FEF), paracentral lobule, Inferior Parietal Gyrus (IPG), lingual gyrus, dorsolateral prefrontal cortex (dlPFC) and supplementary motor area (SMA); the right superior marginal gyrus (SMG). Compared with the NH group, the PC group showed significantly increased ALFF in the bilateral inferior temporal gyrus (ITG) and the left Heschl’s gyrus (HG), superior temporal gyrus (STG) and middle temporal gyrus (MTG).

The comparisons between PC and NH groups showed some similarities in the two frequency bands, such as decreased ALFF in the bilateral precuneus and the right posterior cingulate cortex (PCC), as well as increased ALFF in the right ITG in the PC patients in both bands (Figure S6). In contrast, some obvious differences also existed between the two bands. There was decreased ALFF in the bilateral putamen; the right SOG, AG, SMG, FEF and paracentral lobule; the left PCC, IPG, dlPFC and SMA, along with increased ALFF in the left HG in PC patients compared to the controls in slow-4 band, changes which were not seen in the slow-5 band (Figure S6).

**4. Functional connectivity (FC) results without covariates factors**

The results of the FC group comparisons are presented in Figure S7. Compared with the NH group, the dlPFC showed stronger FC with the temporal pole, STG, cuneus, middle occipital gyrus (MOG), precentral gyrus, SMA and inferior frontal gyrus (IFG) in patients with PC.

**5. ALFF results in full band with covariates factors**

The differences between the PC and NH groups in full band (0.01-0.1Hz) were assessed by the two-sample t-tests. The significance level was set at an FDR-corrected p < 0.01, cluster size > 5 voxels. Age, sex and education levels were imported as covariates. Compared with the NH group, the PC group showed significantly decreased ALFF in the bilateral posterior cingulate cortex (PCC), precuneus, the right superior occipital gyrus (SOG), angular gyrus (AG), frontal eye field (FEF) and the left supplementary motor area (SMA) (Figure S1 and Table S1). Compared with the NH group, the PC group showed significantly increased ALFF in the bilateral inferior temporal gyrus (ITG) and the left Heschl’s gyrus (HG) (Figure S1 and Table S1).

In contrast, some obvious differences existed between full band and slow-4 or slow-5 band. Specifically, there was decreased ALFF in the left IPG, dlPFC and putamen in PC patients compared to the controls in slow-4 band, but these changes were not seen in the full band (Figure S1 and Table S1). There was decreased ALFF in the right AG, SMG, putamen, FEF and paracentral lobule; the left PCC and SMA, along with increased ALFF in the left HG and ITG in PC patients compared to the controls in full band, but these changes were not seen in slow-5 band (Figure S1 and Table S1).

**6. Correlations between Hearing Loss and Cognitive Function**

Partial correlation analyses were used to explore the correlations between cognitive function and PTA or SRT in the PC and NH groups controlling for age, sex and education level. In the PC group, partial correlation analyses revealed that PTA was negatively correlated with AVLT (r = -0.438, p = 0.002) and SDMT (r = -0.396, p = 0.005) and positively correlated with TMT-B (r = 0.355, p = 0.013). SRT was negatively correlated with AVLT (r = -0.323, p = 0.025) and SDMT (r = -0.325, p = 0.024). In the NH group, no correlations were observed between the audiological status and cognitive function.

**Table S1 The difference of ALFF in full band between PC and NH groups**

|  | | **Brain** | **Brodmann** | **MNI coordinates** | | | **T** | **Cluster** |
| --- | --- | --- | --- | --- | --- | --- | --- | --- |
|  | **region** | **area** | **x** | **y** | **z** | **value** | **size** |  |
| **PC > NH** |  |  |  |  |  |  |  |  |
|  | **R Inferior Temporal Gyrus** | 20 | 42 | -39 | -30 | -5.3641 | 46 |  |
|  | **L Inferior Temporal Gyrus** | 20 | -48 | -39 | -24 | -4.3653 | 11 |  |
|  | **L Heschl Gyrus** | 41, 42 | -36 | -24 | 12 | -5.1521 | 14 |  |
| **NH > PC** |  |  |  |  |  |  |  |  |
|  | **R Putamen** | - | 24 | 9 | -6 | 4.9448 | 23 |  |
|  | **L Precuneus** | 7 | -15 | -54 | 15 | 7.9011 | 111 |  |
|  | **R Posterior Cingulate Cortex** | 23 | 21 | -51 | 18 | 9.2654 | 163 |  |
|  | **R Superior Occipital Gyrus** | 18 | 27 | -78 | 12 | 5.6987 | 32 |  |
|  | **R Angular Gyrus** | 39 | 51 | -57 | 36 | 6.3401 | 45 |  |
|  | **L Posterior Cingulate Cortex** | 31 | -6 | -48 | 27 | 4.6735 | 17 |  |
|  | **R Precuneus** | 7 | 9 | -69 | 51 | 4.2048 | 12 |  |
|  | **R Superior Marginal Gyrus** | 40 | 54 | -45 | 39 | 5.6173 | 19 |  |
|  | **R Frontal Eye Field** | 8 | 21 | 30 | 48 | 6.4311 | 27 |  |
|  | **L Supplementary Motor Area** | 6 | -12 | -6 | 66 | 7.2428 | 61 |  |
|  | **R Paracentral Lobule** |  | 6 | -21 | 72 | 4.3406 | 13 |  |

FDR corrected p < 0.01, cluster size > 5 voxels. Abbreviations: ALFF, amplitude of low-frequency fluctuation; PC, presbycusis; NH, normal hearing controls; MNI, Montreal Neurological Institute; L, left; R, right.

# Supplementary Figures


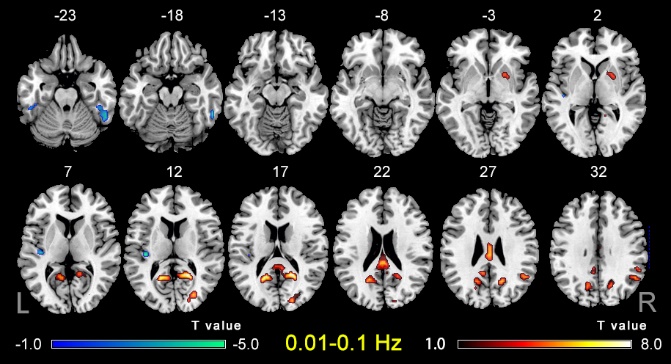


**Figure S1.** The difference of ALFF between the presbycusis (PC) and normal hearing controls (NH) groups in full band (0.01-0.1 Hz). Hot and cold colors indicate significantly higher and lower ALFF in the PC group than in the NH group, respectively. Results obtained by a two-sample t-test. FDR corrected p < 0.01, cluster size > 5 voxels. Abbreviations: L, left; R, right; ALFF, amplitude of low-frequency fluctuation.


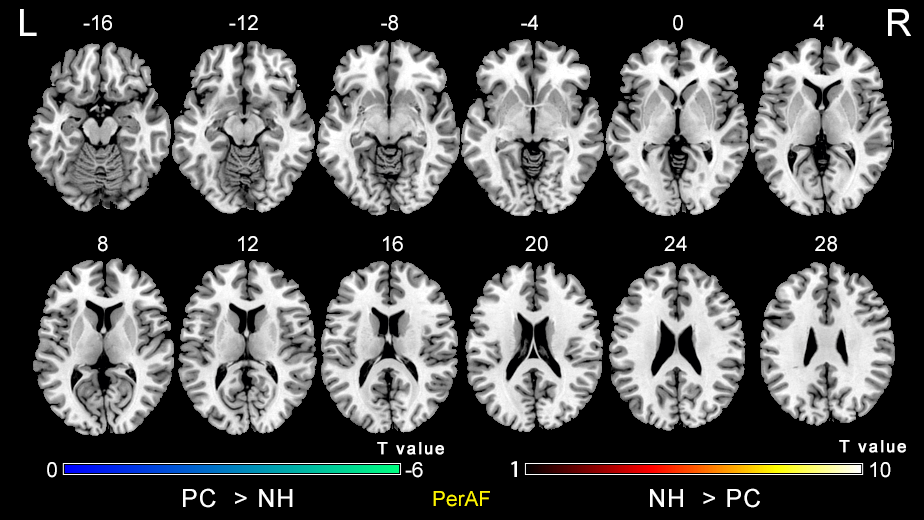


**Figure S2.** Main effect of the group factor on PerAF. There were no significant main effects of group from the two-way repeated-measure ANOVA, FWE correction p < 0.05, cluster size > 5 voxels. Abbreviations: L, left; R, right; PerAF, percent amplitude of fluctuation; ANOVA, analysis of variance.


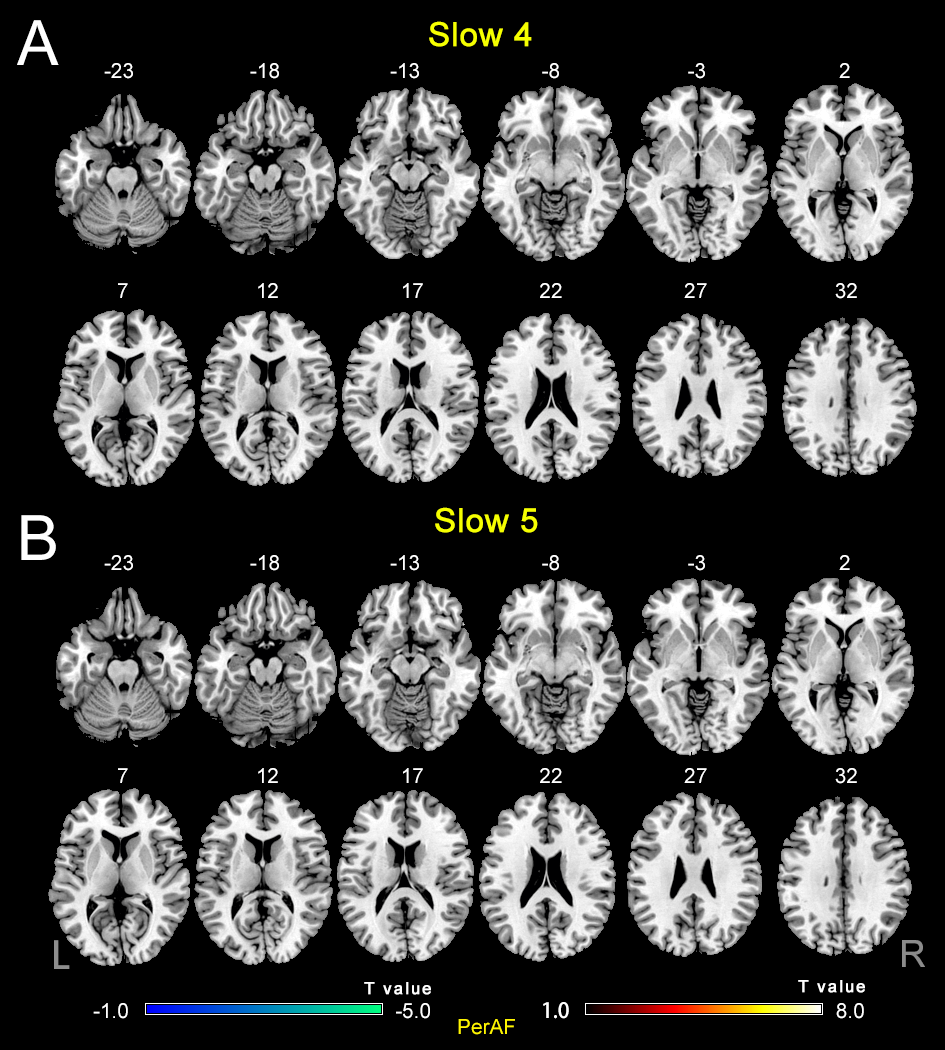


**Figure S3. A:** The difference of PerAF between the presbycusis (PC) and normal hearing controls (NH) groups in slow-4. **B:** The difference of PerAF between the PC and NH groups in slow-5. There were no significant between-group differences in the slow-4 or slow-5 band. Results obtained by a two-sample t-test. FDR corrected p < 0.01, cluster size > 5 voxels. Abbreviations: L, left; R, right; PerAF, percent amplitude of fluctuation.


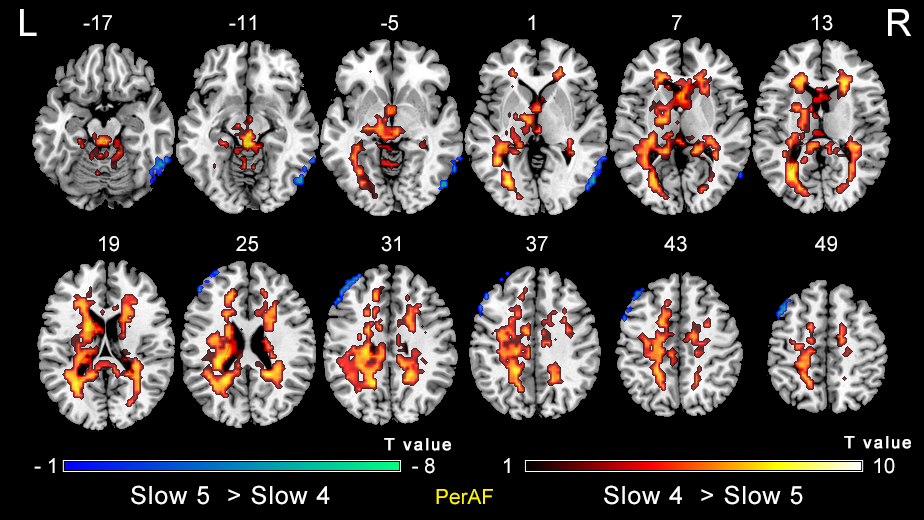


**Figure S4.** Main effect of the frequency band factor on PerAF. Hot and cold colors indicate significantly higher and lower PerAF in the slow-4 band than in the slow-5 band, respectively. Results obtained by a two-way repeated-measures ANOVA. FWE correction p < 0.05, cluster size > 5 voxels. Abbreviations: L, left; R, right; PerAF, percent amplitude of fluctuation; ANOVA, analysis of variance.


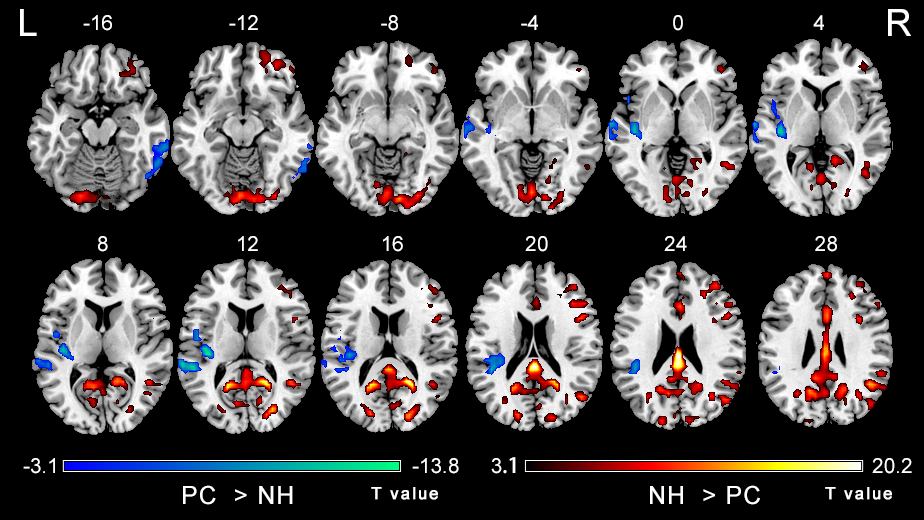


**Figure S5.** Main effect of the group factor on ALFF. Hot and cold colors indicate significantly higher and lower ALFF in the presbycusis (PC) group than in the normal hearing controls (NH) group, respectively. Results obtained by a two-way repeated-measures ANOVA. FWE correction p < 0.05, cluster size > 5 voxels. The results were not taken the age, gender and education as covariates. Abbreviations: L, left; R, right; ALFF, amplitude of low-frequency fluctuation; ANOVA, analysis of variance.


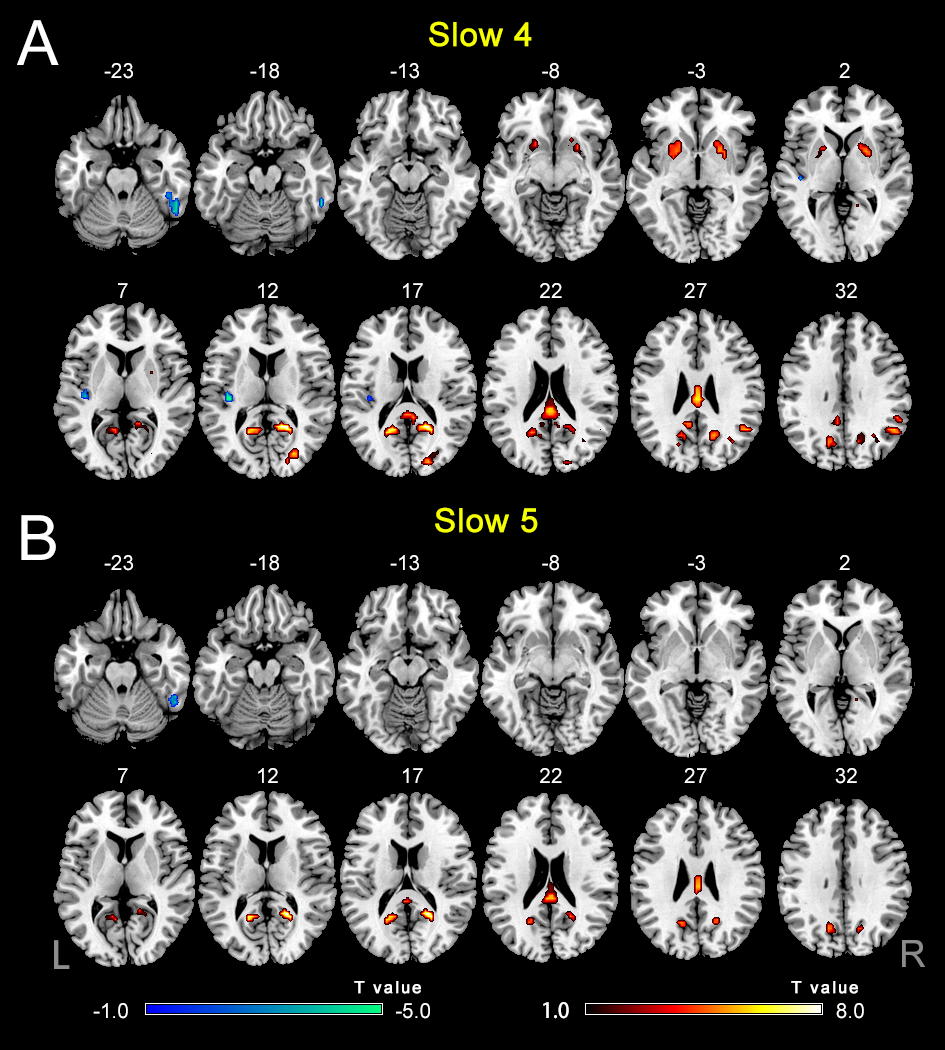


**Figure S6. A:** The difference of ALFF between the presbycusis (PC) and normal hearing controls (NH) groups in slow-4. **B:** The difference of ALFF between the PC and NH groups in slow-5. Hot and cold colors indicate significantly higher and lower ALFF in the PC group than in the NH group, respectively. Results obtained by a two-sample t-test. FDR corrected p < 0.01, cluster size > 5 voxels. The results were not taken the age, gender and education as covariates. Abbreviations: L, left; R, right; ALFF, amplitude of low-frequency fluctuation.


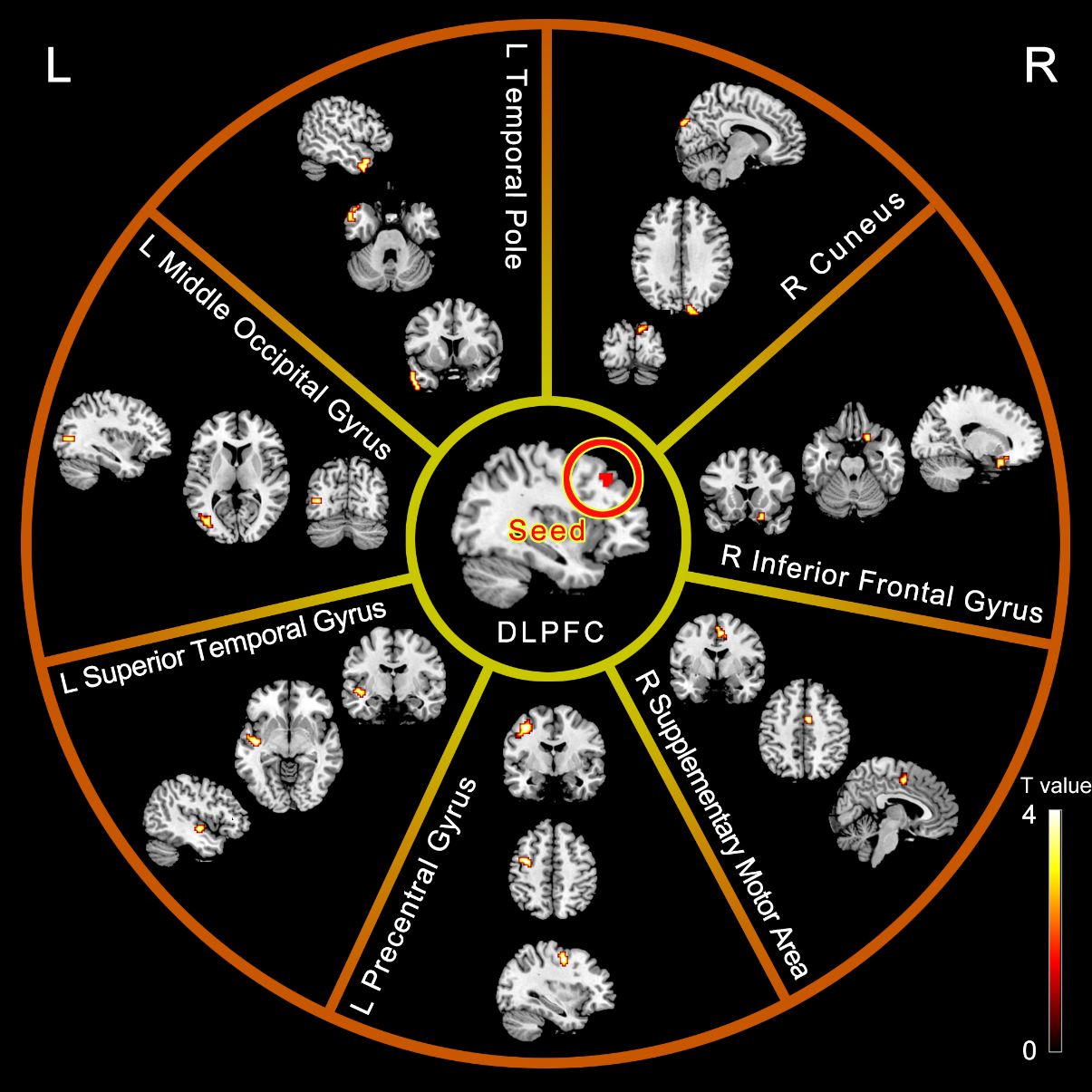


**Figure S7.** ALFF and related FC differences between the presbycusis (PC) and normal hearing controls (NH) groups in slow-4. Between-group differences in FC analyses were only found related to the seed of left dlPFC. Hot color indicates significantly higher FC in the PC group than in the NH group, respectively. Results obtained by a two-sample t-test. FDR corrected p < 0.05, cluster size > 20 voxels.

The results were not taken the age, gender and education as covariates. Abbreviations: L, left; R, right; ALFF, amplitude of low-frequency fluctuation; FC, functional connectivity; dlPFC, dorsolateral prefrontal cortex.

# References

Jia, X.Z., Ji, G.J., Wei, L., Lv, Y.T., and Zang, Y.F. (2017). Percent amplitude of fluctuation: a simple measure for resting-state fMRI signal at single voxel level.

Yang, Liheng, Chen, Qiaohong, Wang, Lingzhen, et al. (2019). Altered Amplitude of Low-Frequency Fluctuations in Inactive Patients with Nonneuropsychiatric Systemic Lupus Erythematosus. *Neural plasticity* 2019**,** 9408612-9408612.

Zhao, N., Yuan, L.X., Jia, X.Z., Zhou, X.F., and Zang, Y.F. (2018). Intra- and Inter-Scanner Reliability of Voxel-Wise Whole-Brain Analytic Metrics for Resting State fMRI. *Frontiers in Neuroinformatics* 12**,** 54.
